# Teaching evidence-based practice to physiotherapy students in Italy: a cross sectional study

**DOI:** 10.1186/s40945-023-00174-5

**Published:** 2023-10-02

**Authors:** Leonardo Piano, Alessandro Chiarotto, Marco Mascarello, Andrea Turolla, Simone Cecchetto, Silvia Gianola, Greta Castellini

**Affiliations:** 1 Unit of Rehabilitation and Functional Recovery, Fondazione Dei Santi Lorenzo E Teobaldo, 12050 Rodello, Italy; 2 Department of Human Neurosciences, Sapienza University of Rome, Rome, Italy; 3 Department of General Practice, Erasmus University Medical Center, Rotterdam, The Netherlands; 4 Department of Health Sciences, Faculty of Science, Amsterdam Movement Sciences, VU University Amsterdam, Amsterdam, The Netherlands; 5 Department of Neuroscience and Rehabilitation, University of Ferrara, Ferrara, Italy; 6 Department of Biomedical and Neuromotor Sciences-DIBINEM, Alma Mater Studiorum, Università Di Bologna, 40138 Bologna, Italy; 7 Unit of Occupational Medicine, IRCCS Azienda Ospedaliero-Universitaria Di Bologna, 40138 Bologna, Italy; 8 Direction of Health Professions, APSS, 38123 Trento, Italy; 9 Unit of Clinical Epidemiology, IRCCS Istituto Ortopedico Galeazzi, Milan, Lombardia Italy

**Keywords:** Evidence-based practice, Education, Physiotherapy

## Abstract

**Background:**

Evidence-based practice (EBP) is being rapidly adopted by the Italian physiotherapy community, although a knowledge gap persists at clinical level with consequent lack of integration of EBP into ground roots practice. Teaching of EBP during the Bachelor of Science (BSc) undergraduate course in physiotherapy likely has a vital role to play in the spread of knowledge, providing a grounding in the fundamental concepts of EBP.

The aim of the present study was to investigate the prevalence of EBP educational content in Italian BSc courses in physiotherapy.

**Methods:**

This is a cross-sectional study during which characteristics of EBP teaching in BSc degree courses of physiotherapy in Italy were collected from institutional websites during the period May to September 2021 with an update in August 2022. We used the STrengthening the Reporting of Observational studies in Epidemiology (STROBE) guidelines for our manuscript.

**Results:**

Forty-two physiotherapy BSc degree programs were retrieved, accounting for all the BSc delivered in the 2021–2022 academic year. Fourteen of these (33.3%) did not report EBP content. Northern universities provided EBP content in 16 out of 18 (88%) degree courses. Central Italian universities provided EBP content in 6 out of 9 (66.6%) degree courses. Southern universities delivered EBP content in 3 out of 9 (33.3%) degree courses. The universities of Sicily and Sardinia provided EBP content in 2 out of 5 (40%) degree courses.

The degree courses taught in public universities were more likely to contain EBP material (25 out of 37, 67.4%), compared to those taught within the private system (3 out of 5, 60%).

**Conclusions:**

The prevalence of EBP content within physiotherapy BSc degree programs in Italy can be considered suboptimal, with both regional differences and according to the system (public vs private). The results of this study could be used as a stimulus for increasing investment in the teaching of EBP in Italian physiotherapy degree courses, thereby improving educational standards.


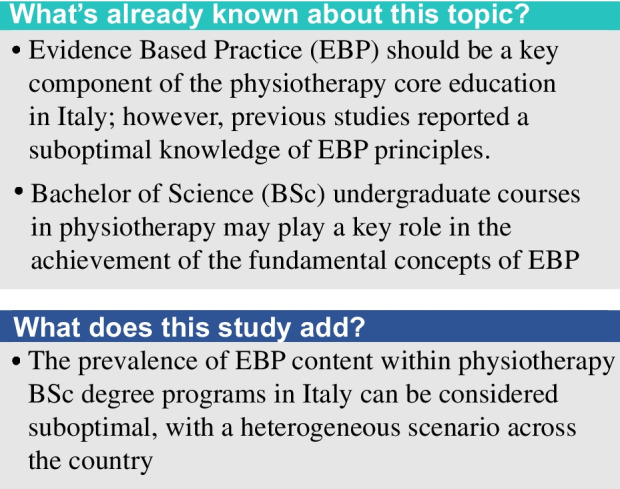


## Background

Evidence-based practice (EBP) is the “conscientious, explicit and judicious use of current best available evidence in making decision about the care of a patient” [1]. From a practical point of view, EBP combines high quality evidence (such as information from studies at low risk of bias), clinical expertise (practitioners’ experience and knowledge) and patients’ values (their attitudes, beliefs and coping strategies), with the aim of improving the quality of health care [2]. Since the beginning of the 1990s EBP has become a key component of the core education of both medical and non-medical health professions [3]. During degree courses in health sciences EBP should be considered as important as more traditional subjects such as anatomy or physiology [3].

Within this context, the world of physiotherapy has tailored the broader concepts of EBP to its specific requirements with the definition of evidence-based physiotherapy [4], supported by institutional organizations such as World Physiotherapy [5]**,** which identifies EBP as a fundamental part of undergraduate degree courses [6]. Although more than twenty-five years have passed since the introduction of EBP, barriers still remain to the universal acceptance of an approach in physiotherapy which is based on solid, evidence-based concepts. Insufficient EBP content during undergraduate education is one of the most obvious obstacles to acquisition of evidence-based knowledge for all health professionals [3, 7–9].

Physiotherapy in Italy is a younger discipline and specialty when compared to other countries [10], but according to the same international standards, has included EBP in its core curriculum and competences statement as one of the eight milestones of professional education [11]. Despite these objectives, a knowledge gap persists which has been investigated by various authors: suboptimal knowledge of the tenets of EBP was found among both undergraduate students and licensed physiotherapists alike [9, 12]. This concerning scenario (insufficient knowledge when compared to the relevant benchmark) is also present in other countries and across specialties: a mismatch between the actual and the ideal is well documented for general medicine and other health professions, regarding both degree programs and use of EBP in daily clinical practice [8, 13, 14].

Recently, reports have hypothesized a crisis regarding EBP credibility, above all within the Italian context: poor attention to EBP teaching and insufficient awareness of the importance of research methodology may be two of the major causes of this crisis [15, 16].

Unless final proof of the crisis are not available and considering recent national legislation considering EBP as the source of information on safety and efficacy of health treatments [17], academic institutions should continue to empower the teaching of EBP during undergraduate degree courses. This is especially important when considering the fact that Italian physiotherapists are professionally certified directly upon receiving their bachelor degree, while secondary degrees like MSc or post-graduate education, are not certified to practice in the national health system.

For these reasons it remains vital that EBP courses are adequately represented in the Italian physiotherapy BSc programs, both within the core curriculum and during the development of clinical skills [11].

To our knowledge, no previous studies investigated the prevalence of EBP content of Italian undergraduate courses in physiotherapy with comparison to current recommendations [11].

The aim of this study is to investigate the prevalence of EBP teaching modules within the Italian BSc physiotherapy programs.

## Methods

### Study design

We conducted a cross-sectional study, using the STrengthening the Reporting of OBservational studies in Epidemiology (STROBE) checklist [18].

### Setting

We designed and conducted this study in the context of the Italian university system. Two authors (LP and MM) independently screened Italian BSc courses in physiotherapy using the institutional website Universitaly—Ministero dell’Università e della Ricerca (MIUR) [19]. In order to create a homogeneous group, we only included those BSc programs with an updated study plan for the 2021–2022 academic year. Since no human subjects were involved, no ethical approval was needed for the study.

### Variables

Two authors (LP and MM) independently extracted the following information: the presence or absence of an EBP module, the presence or absence of any of EBP content and the name of the module in which it was included, the duration of the module, expressed either in hours or in university credits (Crediti Formativi Universitari, or CFUs, corresponding to the European Credit Transfer System, or ECTS, which provides a credit for every 25 h of lectures), the academic year (from first to third), the discipline of scientific sector (DSS) such as Medical Science 48 (MED/48) [20], the type of university (whether public or private) and the geographical area in which the university is situated (Northern, Central or Southern Italy, or the islands of Sicily and Sardinia).

### Data sources

We performed our first web-based search between May and September 2021 across the institutional websites of Italian undergraduate degrees in physiotherapy, with an update between June and August 2022. EBP modules were considered to be included in the programs if the study plan contained one of the following titles: ‘Evidence-Based Medicine’, ‘Evidence-Based Practice’, ‘Evidence-Based Physiotherapy’, “Evidence-Based Rehabilitation” or ‘Evidence-Based Research’. Where specific EBP modules were lacking, we analyzed the presence of ‘EBP content’ in each of the following sections of the curriculum: hygiene, statistics, epidemiology, rehabilitation methodology, professional workshops, additional learning activities and research methodology. The presence of EBP was assigned to those sections of curricula that included the term Evidence-Based Practice or one or more of the related terms listed above. The Eurostat guidelines were used to define the regions of Italy according to geographical position: [21] i) Northern Italy comprises: Aosta Valley, Piedmont, Liguria, Lombardy, Emilia-Romagna, Trentino-Alto Adige/South Tirol, Veneto, and Friuli Venezia-Giulia; ii) Central Italy comprises: Tuscany, Umbria, Marche and Lazio; iii) Southern Italy comprises: Campania, Abruzzo, Molise, Basilicata, Apulia and Calabria); and iv) the major islands are Sicily and Sardinia.

Thus, we collected two data related to the consideration of EBP within the curriculum of studiorum: the amount of CFUs (which are the equivalent of ECTS) and the presence of “specific” teaching course on EBP principles.

### Statistical methods

We reported descriptive statistics as medians and interquartile ranges (IQRs) or absolute values, frequencies and percentages, when appropriate.

## Results

**Fig. 1:**
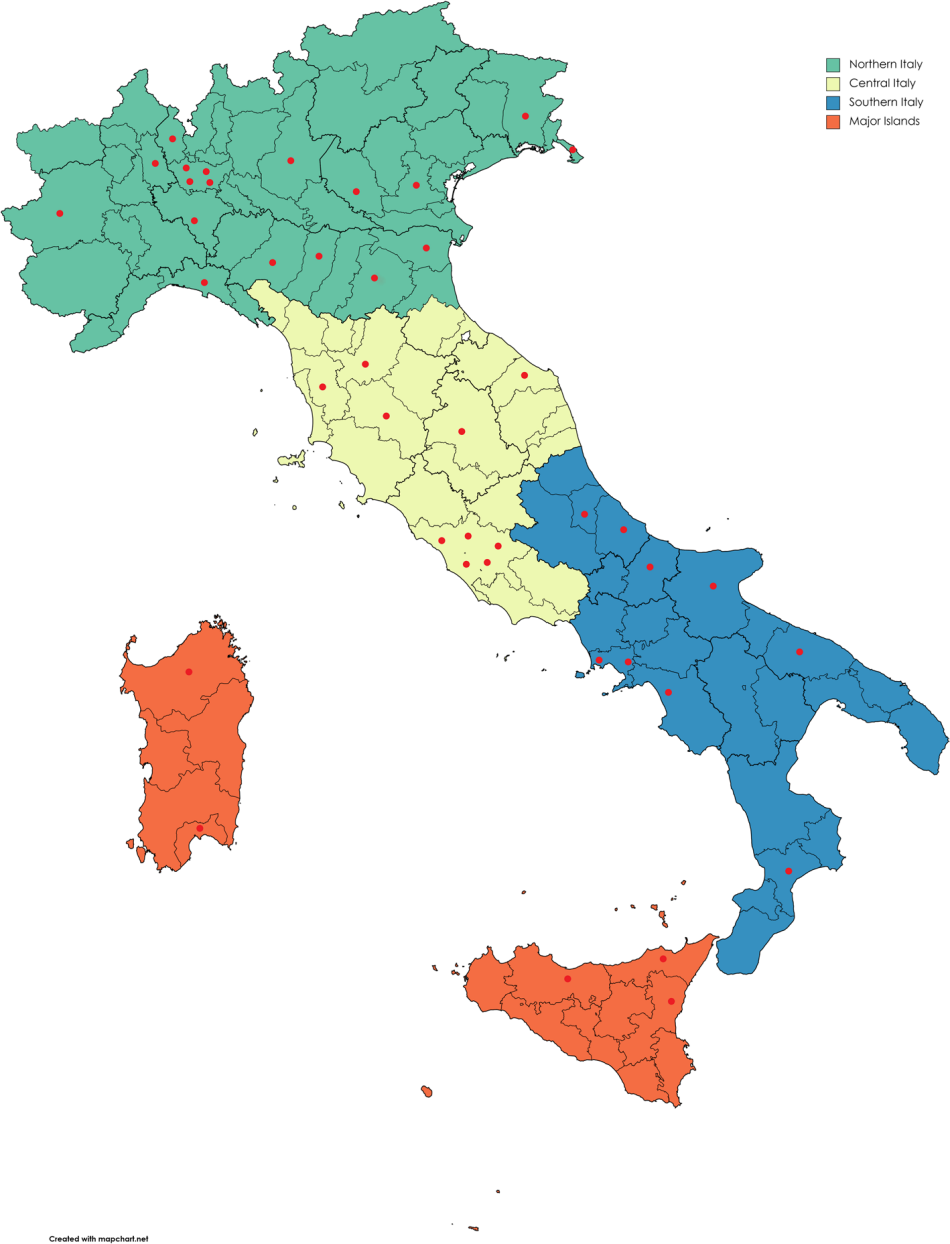
Map of the 42 Italian BSc in physiotherapy. Each red dot corresponds to a BSc in physiotherapy. Green colored regions belong to Northern Italy; yellow colored regions belong to Central Italy; blue colored regions belong to Southern Italy; orange colored regions belong to major islands (i.e. Sardinia and Sicily)

For this study, we explored the plan of study of all 42 undergraduate physiotherapy degree courses delivered in Italy in the 2021–2022 academic year (Fig. 1). Of these, 37 (88.1%) belonged to the public educational system and 5 (11.9%) to the private one. All of these degree courses had a plan of study updated to the 2021–2022 academic year. These 42 degree courses were potentially eligible for 2597 undergraduate students [22].

Overall, there were 18 degree courses (42.8%) in Northern Italy, 10 (23.8%) in the Center, 9 (21.4%) in the Southern and 5 (11.9%) on the major islands (Table 1). EBP content was clearly described under the headings ‘Evidence-Based Medicine’, ‘Evidence-Based Practice’, ‘Evidence-Based Rehabilitation’ or ‘Evidence-Based Physiotherapy’ in the curricula of 9 undergraduate programs, eight of which belonging to the public system. Northern universities provided EBP content in 16 out of 18 (88%) degree courses. Central Italian universities provided EBP content in 6 out of 9 (66.6%) degree courses. Southern universities delivered EBP content in 3 out of 9 (33.3%) degree courses. The universities of Sicily and Sardinia provided EBP content in 2 out of 5 (40%) degree courses (Table 1).

**Table 1:** Main characteristics of the 42 Italian BSc in physiotherapy

University (n of students)	EBP course	Teaching courses with EBP content	DSS	CFUs
Northern Italy				
N1 (80)		Research and management in the Health System	MED/48	2
N2 (80)		Rehabilitation methodology	MED/48	2
N3 (89)	Evidence Based Practice and research methodology		MED/48	2
		Professional education activities	MED/48	1
N4 (65)		Medical statistics	MED/01	2
N5 (40)		Research methodology and management	SECS-S/02	3
N6 (95)	Nr	Nr	-	-
N7 (50)		Medical statistics and research methodology	MED/01	1
N8^a^ (50)		Statistics	MED/01	1
N9^a^ (40)		Statistics ed epidemiology	MED/01	1
N10 (31)		Research methodology	MED/48	1
N11 (110)		Introduction to thesis project	nr	3
N12 (45)		Research methodology	MED/48	1
N13 (40)	Nr	Nr	-	-
N14 (72)		Morphology and function of the human body	MED/48	2
N15 (50)		Epidemiology and Research methodology	MED/01	2
N16 (30)		Preparatory sciences and introduction to research methodology	MED/42	2
N17 (30)	Evidence Based physiotherapy		SECS-S/05	1
N18 (75)	Evidence Based physiotherapy		MED/48	2
Central Italy				
C1 (63)	Evidence-based practice in physiotherapy		MED/48	1
C2 (35)		Research methodology in physiotherapy	MED/01	2
C3 (25)	Nr	Nr	-	-
C4 (50)	Physiotherapy following best practice		MED/48	3
		Methodology for clinical research	MED/01	3
C5^a^ (50)	nr	Nr	-	-
C6^a^ (75)	Evidence-based rehabilitation		MED/34	2
C7 (276)	Evidence-based rehabilitation		MED/48	2
C8 (70)		Professional labs	nr	1
C9^a^ (15)	Nr	Nr	-	-
C10 (25)	Evidence-based physiotherapy and scientific update		MED/48	1
Southern Italy				
S1 (113)	Nr	Nr	-	-
S2 (100)	Nr	Nr	-	-
S3 (75)		Methodology in physiotherapy and rehabilitation	MED/34	2
S4 (75)		Research methodology in physiotherapy	MED/48	1
S5 (30)	Nr	Nr	-	-
S6 (75)	Nr	Nr	-	-
S7 (30)		Medical statistics	MED/01	2
S8 (70)	Nr	Nr	-	-
S9 (45)	Nr	Nr	-	-
Major Isles				
M1 (25)	Nr	Nr	-	-
M2 (38)		Research methodology and epidemiology	MED/01	3
M3 (98)	Nr	Nr	-	-
M4 (35)	Nr	Nr	-	-
M5 (32)	Professional deontology and Evidence Based physiotherapy		MED/48	

*DSS* Discipline Scientific Sector, *CFUs* Crediti Formativi Universitari (i.e. 25 h of teaching for each CFU)

^a^private university, *nr* not reported, *N* Northern Italy University, *C* Central Italy University, *S* Southern Italy University, *M* Major Islands, *MED/01* Medical statistics, *MED/34* Physical medicine and rehabilitation, *MED/42* Hygiene, *MED/48* Nursing and technical neuro-psychiatric and rehabilitation sciences, *SECS-S/02* Statistics for research, *SECS-S/05* Social statistics

Fourteen out of 42 (33%) BSc programs did not provide EBP content in either specific contexts (for example, under the headings of ‘Evidence-Based Medicine’, ‘Evidence-Based Practice’, ‘Evidence-Based Physiotherapy’, ‘Evidence-Based Rehabilitation’ or ‘Evidence-Based Research’) or other teaching courses at postgraduate level (Table 1). Twelve out of these 14 programs (85.7%) were delivered by public universities whereas 2 (14.3%) belonged to the private sector (Table 1). Overall, the BSc in physiotherapy belonging to the public system seem to have a higher prevalence of EBP content (25 out of 37, 67.4%) compared to courses within the private system (3 out of 5, 60%).

Of the courses delivering EBP content (that is, courses specifically in EBP and those containing EBP content), 14 (43.8%) belonged to the MED/48 DSS (nursing and technical neuro-psychiatric and rehabilitation sciences) section, one of the 2 fields of nursing, rehabilitation and psychiatric sciences; 9 (28.1%) EBP courses belonged to the MED/01 section (medical statistics); 4 (12.5%) to MED/34 (physical medicine and rehabilitation); 2 (6.3%) to SECS-S/02 (statistics for research); 1 (3.1%) to SECS-S/05 (social statistics); and 1 (3.1%) to MED/42 (hygiene); whereas it was not possible to attribute 1 (3.1%) to any DSS in particular. University credits gained through modules containing EBP ranged from one (implicating 25 h of course work) to six (150 h course work).

The median number of CFUs was two: 10 out of 28 BSc in physiotherapy had one CFU dedicated to EBP content, three out of 28 BSc had more than two CFUs, and 15 out of 28 BSc had two CFUs.

## Discussion

This cross-sectional study aimed to map the Italian university context to the prevalence of EBP content in undergraduate degree courses in physiotherapy, given that EBP must now be considered an essential part of every health professional’s basic training [3, 11]. To our knowledge, no studies – both in Italy or other countries – have yet explored the current prevalence of EBP content in undergraduate education and few studies have investigated students’ perspectives without collecting data directly from physiotherapy faculties [23, 24]. Taking our hands-on experience as clinical tutors within Italian BSc courses in physiotherapy as a starting point, we hypothesized that EBP content was currently not adequately represented in undergraduate courses in Italy.

We found a substantially low prevalence of EBP content within undergraduate physiotherapy in Italy when compared to both international (World Physiotherapy) and national (Italian Core Competence) recommendations [6, 11]. The education framework advocated by World Physiotherapy deems EBP a milestone of the curriculum studiorum, which requires specific teaching courses (e.g. research methodology) and to be integrated in all the professional (e.g. physical modalities, exercise) [6]. Also, the Italian Core Competence document recommends what a licensed physiotherapist should gain after the BSc education [11]: again EBP has great importance during all the educational pathway.

Nevertheless, EBP content is present in only about 70% of physiotherapy BSc programs, with a heterogeneous scenario across the country.

These findings could have a twofold interpretation: a likely different approach and weight towards the importance of EBP content within the study plan and a certain independence of each university when drafting the plan of studies [25]. As a consequence, these differences might affect the education of Italian physiotherapists, leading to heterogeneous approaches to EBP, with differences in caring for their patients, in accessing to best and updated effective treatments.

Public universities had a higher prevalence of EBP content compared to private ones – although the low number of private universities raised doubts regarding the validity and reliability of these findings to make a meaningful comparison.

Furthermore, these results require extreme attention about their generalizability, since a substantial uncertainty exists regarding the differences between public and private universities, both in Italy and in an international context [26–33].

Moreover, courses dedicated specifically to EBP comprised 21.4% of total BSc courses, while those containing EBP content comprised 50.0% of total BSc courses. Delivery of EBP content was found to be fragmentary not only on a geographical level but also with regards the modality of delivery, the naming of courses and the relevance of EBP (i.e. the number of CFUs dedicated to specific EBP courses or courses with EBP content) within the degree curriculum.

Such a heterogeneous situation has previously been reported within the context of low-income countries [34] but is not what would generally be expected for a high-income country [35].

Even in the presence of adequate education, various other factors including problems of negative attitude, gaps in knowledge and the presence of cultural barriers may limit the implementation of EBP principles. Previous studies have demonstrated that a positive attitude towards EBP was elicited from groups of both licensed physiotherapists and physiotherapy students but that both also declared concerns over theoretical aspects (such as knowledge and skills), organizational ones (perceived lack of time), and technical aspects (such as lack of resources), all leading to reduced adoption of the tenets of EBP in their current, and above all, future clinical practice [7–9, 24, 36–38]. Many students and practitioners reported lack of confidence with the principles and practice of EBP [38] and this is surely a problem that needs addressing. Some studies concluded undergraduate education specifically to be the key element in managing this issue [39, 40], with both academic and clinical tutors well placed to aid their students develop a solid foundation in EBP, including those skills necessary for the adequate integration of EBP into their day-to-day practice, [24, 40]*.* In addition, a positive association between the level of exposure to the principles of EBP and the acquisition of competences by physiotherapy students might also exists: the more the competences on EBP, the more accurate, updated and evidence-based in the health care assistance [39, 41]*.* Previous studies have highlighted a mismatch between knowledge of the principles of EBP among physiotherapists without exploring the root causes of such a mismatch [9, 12]. Our findings could help in understanding the current situation at Italian university level, hypothesizing an inadequate entry-level EBP educational program as the main cause of lack of knowledge among licensed physiotherapists and a key issue limiting optimal application of EBP in clinical practice.

At the moment, there are no other published studies on EBP content of physiotherapy undergraduate courses from other countries: our preliminary findings could be useful for Italian stakeholders, but probably also for a wider context to investigate if this suboptimal presence/reporting of EBP teaching courses is limited to Italian universities or affecting other European (or extra-European) countries as well.

This issue could be very relevant since EBP content should be a milestone of the physiotherapist curriculum, and any lack could impact both the educational pathway and clinical practice.

### Limitations

Our study presents limitations and caution is required to avoid under or overestimation of results. Entry selection criteria for the study were based on the inclusion of declared EBP content within physiotherapy BSc course curricula. We retrieved data from the official website (Universitaly) [19] and then from each university website included in the study. Indeed, educational content can encompass different elements, ranging from the purely theoretical teaching of the concept to the integration and development of critical thinking on practice. This approach may present some limitations in terms of coverage of the educational offer. Fourteen of 42 BSc courses did not report any content or teaching course on EBP: it is therefore possible that some EBP content might have ‘flown beneath the radar’ if it was undeclared on the university websites or included in externally-sourced modules. It is also possible, as some authors have pointed out, that some lecturers might include EBP content during their lessons without this being formally declared at curriculum level [42].

However, Italian universities required professors and lecturers to detail the content of each teaching course, therefore we believe that this choice should provide enough reliable data.

Another limitation could be the “binary” approach to collect information about the presence/absence of EBP content within the plan of study: we are aware that this choice could be simplistic but we considered it as reliable and objective option since other types of data (e.g. characteristics of the teachers, pedagogical approach) would be difficult to retrieve and probably not so informative due to the absence of a reference standard. Additionally, the focus of our study was on the presence of specific EBP courses within curricula studiorium, therefore, given this focus, we believe that our approach is appropriate.

It is important that evidence of poor reporting of university EBP syllabus content be discussed and managed however: we do not expect such a heterogeneous approach to the description of ‘traditional’ course subjects, such as anatomy or physiology.

Definitively, we have no certainties if our findings should be interpreted as a poor reporting, a lack in the educational program, or both about the EBP contents.

### Interpretation

Our preliminary findings suggested that EBP needs to gain in relevance within Italian undergraduate degree courses in physiotherapy, including the way in which teaching in this topic is represented on university websites. Ensuring that EBP becomes part of the core of the physiotherapy BSc should be made a priority: it is only through greater knowledge of EBP at grass roots level that practicing physiotherapists will be to effectively respond to the challenges of their profession in a fast-changing world. Practitioners need knowledge of EBP to critically appraise the evidence about the safety and efficacy of their treatments, increase their professional autonomy and respond to the changing requests of their patients [43–45].

Strengthening the culture of EBP among undergraduate physiotherapy students may also positively affect post graduate education [46]: with higher knowledge of EBP principles, the basic skills could better fit with the process of knowledge translation for both clinical (e.g. musculoskeletal or respiratory rehabilitation) and non-clinical (e.g. epidemiology) specialistic courses [47]. Indeed, the growing number of Italian physiotherapists attending doctoral programs (i.e. PhD) could suggest the need for a development of EBP culture from the beginning of the educational pathway [48, 49].

All universities should ensure that their website is updated regarding the inclusion of EBP courses. The demand for improvement in the standards of EBP inclusion is also consistent with both daily clinical practice and the Italian regulatory system [50]. Recent Italian legislation includes the application of the so-called Gelli-Bianco Law (Legge 24–2017) which defines certain aspects of the legal responsibilities of health professionals according to the adherence – or lack of it—to evidence-based guidelines and recommendations [17]. Both the improvement of efficacy and the reduction of the clinical risks associated with any treatment cannot be separated from the rigorous application of the principles of EBP [2, 51–53].

Methodologies particularly suited to the teaching of EBP might include the examination of case studies or discussions of scientific literature in journal clubs: these tools should be integrated with theoretical coursework to help build the modern and evidence-based physiotherapist [46].

## Conclusions

Italian BSc programs in physiotherapy provide insufficient teaching of EBP, with heterogeneous results across the country. The findings of our study offer an indication of what needs to be done at management, administrative and professorial level within Italian universities to improve the problem. Work is required to both increase EBP content on the undergraduate syllabus and improve the representation of this content on university websites, consistent with international parameters. Future research should explore these findings from different perspectives, such as that of the physiotherapy students themselves, and compare and contrast the Italian context to that of other European countries.

## Data Availability

All data generated or analyzed during this study are included in this published article [and its additional files, https://osf.io/5hr46].
